# Comparison of cardiotoxicity between N-methyl-glucamine and miltefosine in the treatment of American cutaneous leishmaniasis^☆^^☆☆^

**DOI:** 10.1016/j.abd.2021.02.002

**Published:** 2021-05-15

**Authors:** Daniel Holanda Barroso, Ciro Martins Gomes, Antônia Marilene da Silva, Raimunda Nonata Ribeiro Sampaio

**Affiliations:** a Universidade de Brasília, Brasília, DF, Brazil; b Hospital Universitário de Brasília, Universidade de Brasília, Brasília, DF, Brazil

Dear Editor,

N-Methyl glucamine (NMG) is the first therapeutic option for the treatment of American cutaneous leishmaniasis (ACL), but it has many adverse effects, with one of the most serious and feared complications being sudden death, caused by cardiac electrophysiological alterations in the electrocardiogram (ECG) associated to the QT-interval prolongation corrected by the Bazett’s formula (QTc).^1^, ^2^, ^3^

The toxicity of antimonials, the occurrence of disease relapses and resistance to these drugs have stimulated the search for other drugs or more effective therapeutic regimens for the treatment of ACL.^4^ This has resulted in the development of miltefosine (hexadecylphosphocholine), which has shown to be effective against several species of *Leishmania* and other protozoa.^5^, ^6^ The drug acts by activating the programmed cell death mechanism, inhibiting the synthesis of phosphatidylcholine, important for cell membrane synthesis and integrity.^5^

This drug has a teratogenic potential, which requires birth control during treatment and up to two months after its completion.^4^

A retrospective cohort study was performed using data from ECG records of patients treated for ACL (20 mg SbV/kg/day for 20 days for cutaneous leishmaniasis and 30 days for mucosal leishmaniasis or miltefosine (1.3 to 2 mg/kg/day - two capsules a day - for 28 days), followed by a weekly ECG in the Dermatology Service between 2008 and 2013.

The inclusion criteria were: patients treated for ACL, aged between 18 and 85 years and the exclusion criteria were: pregnant women, patients with chronic kidney or liver disease, severe heart disease or other diseases, or receiving drugs that could interfere with the ECG. The patients were not taking any other medications at the time and had not been treated for ACL in the past 6 months.

The included patients were divided into two groups: 1 - treated with NMG; 2 - treated with Miltefosine (M).

The relative risk and the percentage of alterations in the EGC during treatment (heart rhythm and rate, P-wave, QRS complex, RR interval, presence or absence of arrhythmias, and QTc interval) were compared in the groups. For the heart rate, the range between 60 bpm and 100 bpm was considered normal, and the limit of 440 milliseconds for QTc for both sexes (Table 1).^7^

**Table 1:** Relative risks comparing patients treated with NMG and M.

	Bradycardia		QTC >440		Arrhythmias^a^	
	RR (95% IC)	p	RR (95% IC)	p	RR (95% IC)	p
**Day 7**						
NMG	1.14 (0.31–4.23)	0.87	0.25 (0.07–0.94)	0.04	0.85 (0.08–8.74)	0.87
M	1		1		1	
**Day 14**						
NMG	0.53 (0.07–3.66)	0.58	6.58 (0.41–105.36)	0.18	1.93 (0.10–37.44)	0.66
M	1		1		1	
**Day 21**						
NMG	1.94 (0.12–31.74)	0.64	7.22 (0.45–115.33)	0.16	2.94 (0.16–55.31)	0.47
M	1		1		1	
**Day 28**						
NMG	0.36 (0.03–4.54)	0.53	0.54 (0.04–7.25)	0.70	0.13 (0.006–2.86)	0.20
M	1		1		1	

CI, Confidence Interval; RR, Relative Risk.

a Arrhythmias found: isolated ventricular extrasystoles.

The variables were expressed as frequency and the comparison between the groups was done using the Chi-squared test, or Fisher’s exact test when more than 20% of the cells showed an expected frequency < 5. A p-value < 0.05 was considered significant. A multivariate model was employed and the relative risk with a 95% confidence interval was calculated to analyze the intensity of the association between each independent variable and the proportion of adverse effects.

The medical records of 111 patients were analyzed and the epidemiological and electrocardiographic data of 53 individuals were recovered, of which 38 were treated with NMG and 15 with M.

The mean age in the NMG group was 48.4 ± 16.29 years, while in the M group it was 58.4 ± 9.16 years, showing a difference between the groups (p = 0.033). In the NMG group, 23 individuals (60.5%) and in the M group, 7 individuals (46.7) were males (p = 0.37).

On the seventh day of treatment (D7), 11.4% of the members of the NMG group and 33.3% of the M group had QT-interval prolongation. On the fourteenth day (D14), 26.6% of the NMG group had QT-interval prolongation, but none in the M group had it. The trend continued on the twenty-first day (D21), as 35.3% of the NMG group had an altered QTc, while there were no changes in group M. As for the heart rate, 28.9% of patients in the NMG group and 26.6 % of the M group had bradycardia during treatment.

Before treatment, 16 (21%) of the patients in the NMG group and 37.5% of the M group had bradycardia (RR = 0.59; 95% CI 0.17 to 1.99); (p = 0.44). The QTc was altered in 13.51% of the NMG group and 6.66% of the M group (RR = 2.02, 95% CI 0.26 to 15.93; p = 0.5495).

The only significant difference between the groups was demonstrated on the seventh day of treatment when patients treated with M more frequently showed QTc >440 (RR = 0.25; 95% CI 0.07 to 0.94); p = 0.04 (Table 1).

Previous studies have already shown that miltefosine can increase the QT interval during treatment when compared to basal values.^8^ The QT-interval prolongation (QT ≥ 440 ms), which translates into a marked increase in the action potential leading to inhomogeneity of the ventricular electrical matrix, favors the occurrence of reentry phenomena, in addition to favoring early diastolic depolarization and “triggered” activity. This QT-interval prolongation is associated with *Torsades de pointes*, a polymorphic ventricular tachycardia, which can exhibit degeneration into ventricular fibrillation, configuring the arrhythmic mechanism of sudden death.^3^, ^9^

Contrary to the expectations, a greater proportion of patients who used miltefosine had QTc > 440 ms on the seventh day of treatment, but this difference was not maintained in the second and third weeks.

Aging is a factor related to electrocardiographic alterations and the patients in group M had a higher mean age.^10^ However, on the other hand, these alterations were already present in the pre-treatment period, which leads us to believe that age did not play a determining role in the alterations. Another limitation is the performance of multiple tests, which can increase the rate of type 1 errors.^11^

Considering the result that suggests M cardiotoxicity in the context of the current trend in the treatment of ACL with the association of potentially cardiotoxic drugs (antimonials, amphotericin), this finding should be better studied in regimens with drug combinations. This seems to be the first study that demonstrated the presence of ECG alterations caused by M ​​over the course of treatment.^4^ These findings, however, have an exploratory characteristic and deserve to be confirmed by studies with a larger number of patients.

## Financial support

Financial support from the Brazilian Society of Dermatology(Sociedade Brasileira de Dermatologia, SBD) through Funaderme and from the Research Support Foundation of the Distrito Federal(Fundação de Apoio à Pesquisa do Distrito Federal - FAP-DF), number 0193.001447 / 2016.

## Authors’ contributions

Daniel Holanda Barroso: Drafting and editing of the manuscript; collection, analysis, and interpretation of data.

Ciro Martins Gomes: Critical review of the manuscript; collection, analysis, and interpretation of data. Antônia Marilene da Silva: Drafting and editing of the manuscript; collection, analysis, and interpretation of data.

Raimunda Nonata Ribeiro Sampaio: Collection, analysis, and interpretation of data; drafting and editing of the manuscript; critical review of the manuscript.

## Conflicts of interest

None declared.
